# Supplementary material for: A Simple Nomogram to Predict Clinically Significant Prostate Cancer at MRI-Guided Biopsy in Patients with Mild PSA Elevation and Normal DRE
Source: Cancers (Basel). 2025 Feb 23;17(5):753. doi: 10.3390/cancers17050753 (PMC11898869; doi:10.3390/cancers17050753)
Supplement: Supplementary file 1 [file cancers-17-00753-s001.zip › cancers-3374124-supplementary.pdf]

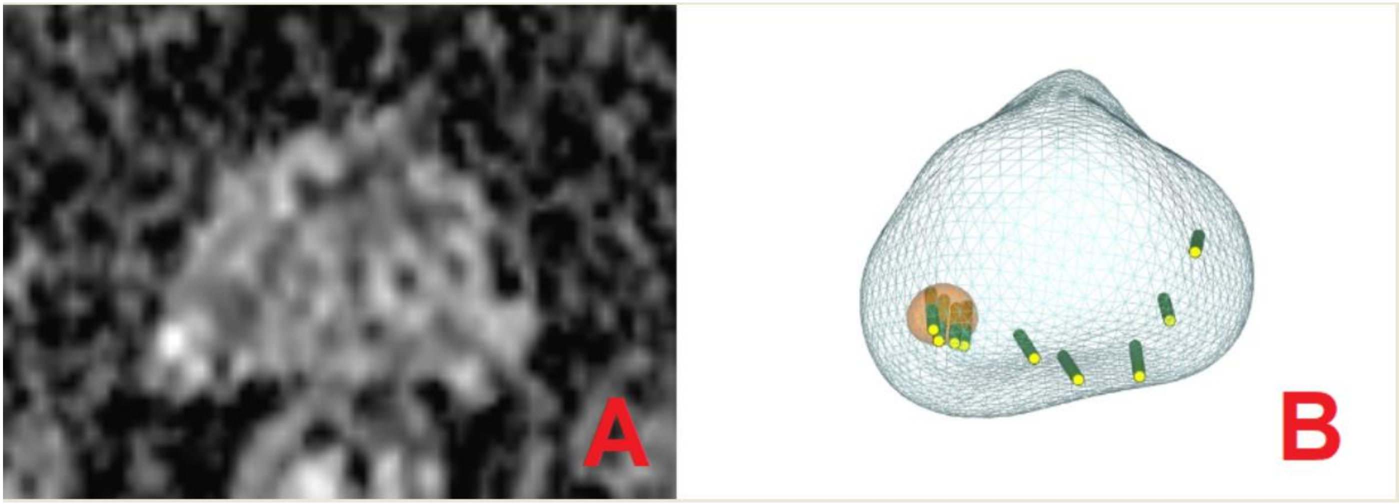

Figure S1: Sample picture documenting an MRI–ultrasound fusion biopsy (the nomogram score for this patient was 76%, he was diagnosed with grade group 2 cancer in targeted cores only).
